# The Progress and Challenges of Implementing HLA Molecular Matching in Clinical Practice

**DOI:** 10.3389/ti.2025.14716

**Published:** 2025-08-13

**Authors:** Suzanne Bezstarosti, Sebastiaan Heidt

**Affiliations:** Department of Internal Medicine, Division of Nephrology and Transplantation, Erasmus MC Transplant Institute, University Medical Center Rotterdam, Rotterdam, Netherlands

**Keywords:** HLA, eplet, kidney transplantation, molecular mismatch, organ allocation

## Abstract

HLA molecular matching in solid organ transplantation in the form of eplets, solvent-accessible amino acids or PIRCHE-II has been proposed as a more granular method than HLA matching on the antigen level. While many studies have shown the association between molecular mismatches and *de novo* donor-specific antibody formation, rejection and graft loss, evidence for prospective molecular matching in allocation is currently lacking, and the actual practical implementation and feasibility of molecular matching remains unclear. In this review the various potential applications of molecular matching in transplantation are discussed, including 1) organ allocation in deceased donor programs, 2) living donor selection, 3) increasing the transplantability of highly sensitized patients and 4) risk stratification to facilitate personalized immunosuppressive management, along with the challenges and gaps in current knowledge regarding these approaches. While clinical application of molecular mismatch analysis in solid organ transplantation holds promise, the fundamentals of HLA-specific antibody biology and epitope-paratope interactions should be further elucidated. This will aid in unraveling the factors that affect the relative immunogenicity of HLA molecular mismatches in order to start using molecular matching in clinical transplantation.

## Highlights

• Molecular mismatch analysis can be used to increase the chance of finding a suitable donor for highly sensitized patients.
• Data regarding eplet frequencies in different populations are required to explore the consequences of eplet-based allocation algorithms for ethnically diverse populations.
• Antibody verification is essential to identify clinically relevant molecular mismatches to be used for deceased donor allocation.
• For deceased donor allocation, high resolution HLA typing is required at the time of allocation.
• Clinical studies with clear pre-defined outcome measures are required to investigate the application of molecular mismatch analysis in kidney paired exchange programs for living donation.
• The application of molecular mismatch analysis for post-transplant risk stratification to guide tailored immunosuppression should be investigated in prospective clinical trials.
• The fundamentals of HLA-specific antibody biology and epitope-paratope interactions need to be further elucidated in order to unravel the factors that affect the relative immunogenicity of molecular mismatches.

## Introduction

**FIGURE 1:**
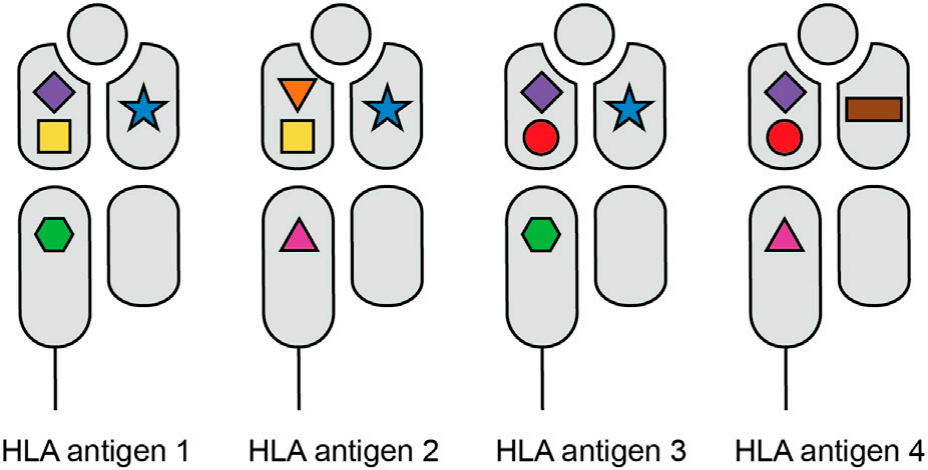
HLA polymorphism on the level of amino acid configurations. The various shapes depict the different amino acid configurations on the HLA molecules. While each HLA antigen expresses a unique set of these polymorphic sites, the individual amino acid configurations can be shared between different HLA molecules.

**FIGURE 2:**
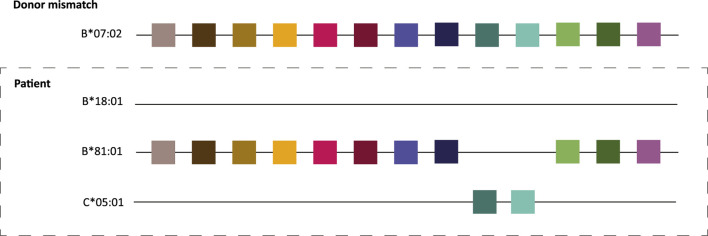
HLA molecular matching principle. The colored squares depict amino acid configurations or eplets present on the donor B*07:02 that are lacking on patient B*18:01. Although there is one HLA antigen mismatch between donor and recipient, there are no mismatches on the molecular level due to shared amino acid patches between the donor allele and the recipient’s HLA B*81:01 and C*05:01 alleles. Adapted with permission from “Principle of HLAMatchmaker is inter and intra-comparison of triplets. In this particular patients all triplets mismatched between HLA-B18 and HLA-B7 are shared by the other HLA alleles of the patient. Final interpretation: no foreign triplets on B18 and therefore no alloantibody induction” by Claas et al. [8], licensed under CC-BY-NC-ND.

After the discovery of the HLA system in the 1950s, Paul Terasaki demonstrated the correlation between HLA matching and kidney allograft survival in 1966 [1]. Since then, HLA matching has been one of the cornerstones of transplantation and transplant organizations across the world have included HLA matching in their algorithms for organ allocation [2], aiming to minimize the chance of rejection and the development of *de novo* donor-specific antibodies (DSA). In Eurotransplant, current HLA matching occurs on HLA-A, -B (broad antigen level), and -DR (split antigen level) only, with priority for full-house matches, and a point system for all other instances [3]. However, due to the high polymorphism of HLA and the scarcity of donor organs, the majority of kidney transplant recipients receive a graft with a certain degree of HLA antigen mismatch [4]. Refinement in HLA typing techniques and amino acid sequence alignments have demonstrated that the high level of polymorphism of HLA can be explained by a few hundred polymorphic amino acid configurations, which are often referred to as epitopes [5, 6], despite this actually being a faulty use of nomenclature [7]. While these amino acid configurations can be shared between different HLA molecules, every individual HLA molecule is comprised of a unique set of these polymorphic sites (Figure 1). In theory, this means that an HLA antigen mismatched graft could be actually fully matched on the level of amino acid configurations (Figure 2), which is referred to as molecular matching. In 2006, Rene Duquesnoy introduced the term “HLA eplet” to describe a configuration of antibody accessible polymorphic amino acids within a 3.5 Ångstrom radius on the HLA molecule, that can be recognized by a B cell receptor through interaction with the CDR-H3 region [9]. Since then, various methods have been developed for HLA molecular match analysis, but HLA eplets, as described in the HLA Eplet Registry and incorporated in HLAMatchmaker [10], remain most well-known. HLA molecular matching has been proposed as a more feasible method to prevent *de novo* DSA (dnDSA) formation than HLA matching on the antigen level [6, 11, 12], and many studies have shown the association between eplet mismatches and dnDSA formation, rejection and graft loss [13–17]. However, since evidence for a beneficial effect of prospective eplet matching in allocation is currently lacking, the actual practical implementation and feasibility of molecular matching remains unclear. In fact, the application of eplet matching in transplantation has even been deemed premature and the question has been raised whether molecular matching will actually reduce the complexity of HLA matching [7]. In this review the various potential applications of molecular matching in transplantation will be discussed, including 1) organ allocation in deceased donor programs, 2) living donor selection, 3) increasing the transplantability of highly sensitized patients and 4) risk stratification to facilitate personalized immunosuppressive management, along with the challenges and gaps in current knowledge regarding these approaches.

## Identification of Clinically Relevant Eplets Is Required for Clinical Application of HLAMatchmaker

The lack of empirical evidence for clinically relevant eplets is the one of the main obstacles for implementation of eplet matching in transplantation. As the current eplet repertoire has been theoretically defined based on HLA amino acid sequences and not on proven immunogenicity, the question remains which eplet mismatches are immunogenic and which are permissible [7, 18]. The identification of clinically relevant eplets is a crucial condition for the modification of allocation algorithms towards eplet-based matching, as denial of an organ offer based on eplet mismatches which are not clinically relevant would be unacceptable.

Antibody verification is currently the only method to validate that specific polymorphic amino acids (translated to eplets) can actually be bound by antibodies. With the lack of better techniques, this measurement of antigenicity is translated to presumed immunogenicity. Much experimental evidence has been gathered over the years, which has been summarized in the HLA Eplet Registry, an online database of HLA eplet data [19–21]. A recent review of the HLA Eplet Registry provides insight in the different methods that have been used for antibody verification, and demonstrates that not all eplets considered antibody-verified by the HLA Eplet Registry were verified based on high quality peer-reviewed research [22], which has prompted an adaptation of the Registry. It also showed that especially for HLA class II, there are several theoretical eplets for which no antibody verification has been performed yet. Therefore, antibody verification of eplets using human HLA-specific recombinant monoclonal antibodies (mAbs), and adsorption/elution studies is still an ongoing and useful effort [23–27].

Antibody verification of eplets using mAbs and eluted antibodies after adsorption from serum is based on the identification of uniquely shared amino acids present on the reactive HLA alleles in the single antigen bead (SAB) assay. However occasionally, even for mAbs, the reactivity analysis in the SAB assay leads to the identification of multiple uniquely shared amino acids that are not located within 3.5 Ångstrom from each other, and therefore cannot be part of a single eplet. Since in these cases the amino acid residues involved are simultaneously present on all reactive HLA alleles in the SAB kits, it is not possible to determine which of these residues are truly crucial for antibody binding. Site-directed mutagenesis of wildtype HLA molecules is an elegant strategy to tackle this problem and has been recently used to narrow down an HLA-A1-specific antibody reactivity pattern consisting of three distant amino acids to a single amino acid [28]. Additionally for HLA class II, CRISPR-Cas9 modified cells have provided insight in crucial regions for the binding of an HLA-DQ specific antibody with its target HLA [29].

One of the exciting next steps in further understanding the fundamental biology of HLA antigen and antibody interaction, is the actual visualization of antibody binding to its target HLA using crystal structures or cryogenic electron microscopy (cryo-EM). Such studies can further inform on the correctness of eplet definition and verification. Moreover, these approaches will increase our understanding of the electrostatic and physiochemical properties of amino acids that are relevant for immunogenicity [30–32]. Recently, the first crystal structure of an HLA-A*11:01-specific antibody bound to its target HLA has been reported [33]. The characterization of the epitope-paratope interaction demonstrated that the single amino acid that was predicted to be crucial for antibody binding, was indeed part of the epitope. Importantly, while this study showed the power of crystal structure analysis, the described HLA-A*11:01-specific antibody was generated using a phage library, and may not represent an antibody developing during a human immune response to mismatched HLA. Currently, studies are ongoing to characterize the binding of fully human HLA-DR and -DQ-specific mAbs using cryo-EM, which allows for visualization of the binding of these mAbs to their target molecules. A comprehensive overview on epitope-paratope biology and the impact on antibody binding has been published elsewhere [34].

Since antibody verification of eplets using human mAbs and adsorption/elution studies on serum samples is a slow and laborious process, ideally a prediction algorithm of HLA epitope immunogenicity should be developed. Preferably, this algorithm would incorporate all existing evidence generated by the reactivity patterns of HLA-specific mAbs, the identified crucial amino acids by mutagenesis and crystallography or cryo-EM, and the physiochemical and electrostatic properties of the amino acids involved. Additionally, the role of T cell epitopes in the humoral alloimmune response has to be investigated further, as T cell help is required for the initiation of a long-lived antibody response [35]. Although predicted T cell epitopes (PIRCHE-II) have been associated with graft failure in kidney transplantation [36], the approach of predicting HLA-derived T cell epitopes presented in recipient HLA class II molecules is at the moment merely based on the underlying amino acid differences between HLA alleles and relatively low peptide binding affinity to HLA class II. Currently, there is no experimental evidence on whether the predicted peptides derived from allogeneic HLA are actually generated in the lysosomal compartment. Furthermore, by setting a relatively low binding strength threshold, it remains to be determined which predicted peptides are actually presented by HLA class II molecules *in vivo.*

Ultimately, a comprehensive model of HLA immunogenicity for the induction of a humoral immune response should incorporate the antigen-specific crosstalk between B cells and T cells. Molecular mismatch scores on the B and T cell level have been combined in several studies, hinting towards clinical relevance of taking both arms of the immune system into consideration [37–39]. However, these studies have merely combined molecular mismatch scores, irrespective of which specific allele these disparities occurred. Studies where true linked recognition between B cells and T cells is taken into consideration are currently lacking.

Finally, the level of immunogenicity of individual eplets is not merely based on amino acid mismatches, their physiochemical properties and the availability of T cell help, but also dependent on the population that is investigated. This calls for studies that investigate the differential immunogenicity of eplets in large datasets of various and ethnically diverse populations. The differential immunogenicity of molecular mismatches across transplant populations worldwide is one of the topics of the 19th International HLA & Immunogenetics Workshop that will be organized in 2026.

## Deceased Donor Allocation

To date, only one small pilot study from 2016 has prospectively investigated incorporating eplet mismatch loads into deceased donor allocation for 19 pediatric kidney transplant patients. By determining individual eplet mismatch thresholds for each patient at the time of listing, recipients received a significantly lower number of HLA Class II eplet mismatches compared to the general allocation scheme [40]. While the numbers were small, low rates of dnDSA within the first 12 months are promising. However, a major limitation in the chosen strategy is that high-resolution typing of deceased donors at the time of allocation was not available, which poses one of the major obstacles on the road towards implementation of molecular matching in transplantation. For proper molecular mismatch analysis for the individual patient, high-resolution HLA typing of both the donor and potential recipient(s) is required. While many transplant centers have introduced high-resolution typing for the living donor transplantation setting, high-resolution typing is not yet routinely performed for deceased donor transplantation in most transplant centers. Importantly, HLA typing on the second field level is not only more costly than low-resolution typing, but also takes more time to complete. Most commercial kits offering high-resolution typing based on next-generation sequencing take 1–5 days for completion [41], making it an unsuitable technique for the typing of deceased donors. Nonetheless, a recent study described a high-resolution typing method using Nanopore sequencing, which resulted in high-resolution typing for 11 loci within 4 h, indicating that second field typing for deceased donors within the timeframe of allocation is within reach [42]. Several commercial companies are currently optimizing the Nanopore sequencing workflow for deceased donor typing.

A provisional solution for the lack of high-resolution typing is imputation of second field typing based on low-resolution haplotypes [43]. This method has been applied frequently in retrospective cohort studies investigating molecular mismatch association with transplant outcomes. In the absence of true high-resolution HLA typing data, this approach is justifiable for large retrospective studies. However, as this method can lead to inaccuracies in molecular mismatch estimations, it is not deemed suitable for individual patients in the clinical setting [44, 45].

Secondly, as HLA allele frequencies vary considerably amongst different populations in the world [46], this inherently means that frequencies of specific molecular mismatches will differ across different populations. Eplet repertoire variation has to be taken into account in studies that investigate differential immunogenicity of individual eplets, because a very high or very low frequency of a given eplet in a population can skew immunogenicity scores, which may consequently not be applicable to other populations [47]. The fact that this issue needs consideration is illustrated by the situation in the United States, where it became clear that African Americans were disadvantaged regarding access to kidney transplantation due to HLA matching requirements [48]. Subsequently, priority for HLA-A and HLA-B matching was eliminated in the kidney allocation system of the United Network of Organ Sharing [49, 50]. Data regarding eplet frequencies in different populations are required, so that the consequences for the implementation of eplet-based allocation algorithms for ethnically diverse populations can be investigated. In this light, a recent study investigated eplet frequencies across six different ethnic groups using HLA alleles included in the Common, Intermediate and Well-Documented (CIWD) 3.0.0 catalogue and demonstrated that 98.6% of eplets are present on the common HLA alleles in all ethnic groups [51]. It should be noted however that HLA allele frequencies (and thus eplet frequencies) may be different in donor and recipient populations.

Related to the issue of equity is the concern that molecular matching would lead to longer waiting times on the transplant waiting list, especially for ethnic minorities. As quality of life is poor for patients on dialysis, and the survival benefit of transplantation as compared with dialysis is significant [52], it would not be acceptable that improved HLA compatibility by utilizing molecular matching would be at the expense of longer waiting times. Currently, data are scarce on the consequences of molecular matching for the kidney transplant waiting list regarding waiting times and equity for ethnic minorities. A recent simulation study from the United States suggested that eplet-based allocation prioritizing HLA-DQ eplet matching was equitable for Black and Hispanic candidates, but this was not the case for Asian patients [53]. Additionally, a Canadian simulation study in a highly ethnically diverse population demonstrated that eplet matching would allow for better matched grafts by converting alleles to eplets, and that eplet matching would be feasible even within a waiting list of only 250 patients [54]. However, neither of these studies included simulations regarding waiting time. An allocation simulation study investigating the effect of T cell epitope matching on waiting times in the Eurotransplant region found that this approach did not significantly impact waiting times [55].

## HIGHLY SENSITIZED PATIENTS AND RETRANSPLANTATION

Although the road to implementation of molecular matching in deceased donor allocation algorithms still seems long, molecular mismatch analysis can already be utilized to increase the chance of finding a suitable donor for highly sensitized patients. Since the chance of finding a donor for these patients is very slim due to the large number of unacceptable antigens, the Acceptable Mismatch (AM) Program was launched in the Eurotransplant region in 1989 [56]. Unlike regular allocation, the AM program is based on finding a donor that is compatible with the patient’s HLA type plus acceptable antigens, which are defined as antigens to which the patients has not developed antibodies [57]. Defining additional acceptable antigens based on triplet sharing was in fact the primary concept behind HLAMatchmaker, as described in the early 2000s by Rene Duquesnoy and Frans Claas [58, 59]. In the present day of SAB technology, eplet analysis can be utilized for the definition of acceptable antigens: by analyzing SAB data from the highly sensitized patient, eplets present on antigens towards which no antibodies have been formed can be extrapolated to antigens not tested in SAB assays, to maximize the number of acceptable antigens defined [58]. Furthermore, eplet analysis can assist in determining which of the unacceptable antigens as established by SAB analysis can be explained by a previous immunizing event (such as a previous transplantation, pregnancy or blood transfusion), and are therefore truly unacceptable. If the reactivity cannot be explained by an immunizing event or a shared eplet thereof, this bead reactivity might be background or non-specific binding. Such information should prompt further investigation of the reactivity (for instance using flow-cytometry cross match), in order to decide whether this antigen should be listed as unacceptable, or rather be considered as a risk factor, taking into account MFI value of the reactive antigen [34]. A recent study from Portugal showed that calculation of an eplet-based virtual PRA increased the transplant probability for highly-sensitized patients [60].

In line with the aforementioned, a recent study investigated patients with pre-existing so-called “donor epitope-specific antibodies (DESA)” and showed that clinically relevant DESA were associated with increased risk on graft loss in deceased donor transplantations [61]. Analysis of “molecular mismatch-specific HLA antibodies” might be of particular interest in patients that undergo retransplantation, as shown in a recent study where retransplant patients with pre-existing DSA that target a repeated molecular mismatch (i.e., antibody verified eplets), had lower graft survival and higher ABMR rates than patients with DSA that were not directed against a repeated molecular mismatch [62]. Data on the clinical relevance of repeated HLA mismatches in retransplant candidates are scarce. A recent study demonstrated that repeated HLA‐DRB1 and HLA‐DQB1 mismatches on the split antigen level in the absence of pre-existing DSA affected DSA formation, rejection and graft survival [63]. Unfortunately, no analysis on the molecular mismatch level was performed. A case report of a female transplant recipient that was highly sensitized by three pregnancies described that she developed ABMR due to a repeated eplet mismatch between her husband and her donor [64]. More studies investigating the clinical relevance of repeated mismatches on the molecular mismatch level are required.

## Living Donation

**FIGURE 3:**
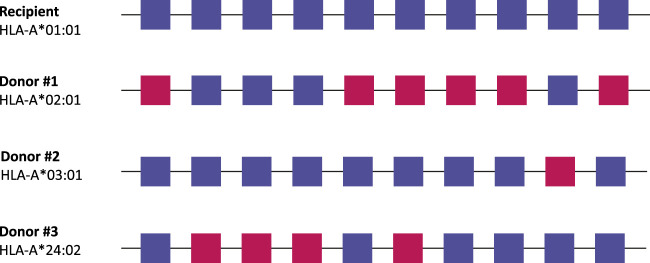
Living donor selection based on molecular mismatch levels. In this example, every potential donor has a single HLA antigen mismatch with the recipient, but a different number of antibody-verified eplet mismatches. In this case, donor #2 would be the best option for the recipient, as there is only one antibody-verified eplet mismatch with the recipient. Antibody-verified eplet mismatches are assessed taking into account the full HLA typing of the recipient. Squares: antibody-verified eplets. Blue, matched eplets; red, mismatched eplets.

While the previous section was predominantly related to deceased donor transplantation, determining the molecular mismatch level can also be of value in living donor transplantation. Primarily in patients that are likely to require a retransplantation later in life (*e.g.*, pediatric patients), it is critical to limit the chance of dnDSA formation at the first transplantation, so that there are no pre-existent HLA-specific antibodies that can impede a repeat transplantation at a later timepoint. In cases where there are multiple potential (otherwise comparable) donors, molecular mismatch analysis can inform on which mismatched donor HLA has the lowest chance of inducing an antibody response (Figure 3). Furthermore, molecular mismatch analysis of prospective transplant recipients patients that have a compatible potential living donor could inform clinicians to explore whether entering a kidney paired exchange program might offer a lower immunological risk. To date only one study has prospectively investigated eplet matching in a kidney paired exchange program. In this study, results from seven pediatric patients included in the Australian Kidney Exchange were reported. According to the authors, all patients were transplanted with a lower immunological risk compared to their registered donors. However, three patients did develop DSA within a median follow-up period of 12 months [65]. Furthermore, the National Kidney Registry in the United States, an organization that facilitates hundreds of paired exchange transplantations annually, has been promoting eplet matching on their website for several years [66]. The first results of this program are expected to be published soon.

With such limited data, the use of molecular mismatch analysis in kidney paired exchange programs should further be methodically investigated in clinical studies. Regardless, other factors besides HLA will contribute to the definitive selection of a living donor, including blood group, age, medical history, and psychological and social factors. Which method of molecular mismatch calculation (i.e., eplets, solvent-accessible amino acids or PIRCHE-II) will be most appropriate for donor selection will have to be demonstrated in clinical studies.

## Post-Transplant Risk Stratification

Identification of immunogenic and permissible eplet mismatches is essential for the implementation of molecular matching in deceased donor allocation schemes and molecular mismatch analysis for living donor selection. However, deciphering immunogenicity of individual eplet mismatches may be less relevant for utilizing eplet mismatch levels for post-transplant risk stratification, as eplet mismatches would be merely used as a tool to assess the risk for immunological rejection after transplantation, rather than being incorporated into organ allocation and donor selection.

Many studies have demonstrated that eplet mismatch loads are associated with the risk of DSA formation, rejection and graft loss after transplantation on the cohort level [13–15, 17, 67, 68]. Furthermore, Wiebe et al. have shown that HLA class II eplet mismatch load was associated with the tacrolimus trough levels that are required to prevent DSA formation [69]. In a cohort of 596 kidney transplant recipients, HLA-DR/DQ eplet mismatch was a predictor of dnDSA development and patients with a high number of eplet mismatches were less likely to tolerate low tacrolimus levels without developing dnDSA. As there still is an unmet need for tools that could guide personalized immunosuppressive therapy in transplantation [70, 71], HLA molecular mismatch level in the form of amino acids, eplets or PIRCHE-II could potentially be used as a parameter in post-transplant risk stratification. However, there are several challenges considering the application of molecular mismatch for this purpose.

Firstly, it is unclear which HLA loci should be considered in post-transplant risk stratification. The majority of studies have reported data on HLA class II, since most dnDSA are directed at HLA-DQ mismatches. However, it is not clear if only HLA class II, or even only HLA-DQ should be considered, or that all classical HLA loci should be taken into account (Meziyerh et al., submitted).

Secondly, as eluded above, there are several methods for calculating HLA molecular mismatch loads [34] which all have been associated with transplant outcomes like DSA formation and graft survival. The use of different computer programs, including HLAMatchmaker, PIRCHE-II [36], the Electrostatic Mismatch Score (EMS) [32] and HLA-EMMA [72], makes it difficult to compare studies, because each method will result in different optimal cutoff values and ranges (see Table 1 for an overview of the most used methods). Moreover, the eplet repertoire as analyzed by HLAMatchmaker has changed over the last years, which resulted in different eplet mismatch thresholds in different studies [69]. This heterogeneity impedes the ability to draw conclusions leading to thresholds that can be validated in other cohorts. The vast diversity in the primary outcomes of the aforementioned retrospective studies (ranging from allograft survival at 10 years [73], to ABMR and DSA at 5 years [17] and ABMR, TCMR and dnDSA at 10 years [15]) emphasizes the need for prospective studies with clear pre-defined outcome measures.

**TABLE 1:** Methods for HLA molecular mismatch analysis.

HLAMatchmaker	Excel program that compares the amino acid sequences of donor and recipient HLA alleles to identify mismatched eplets. An eplet is defined as a cluster of polymorphic amino acid residues within a 3.0–3.5 Ångstrom radius.^a^ eplets have been incorporated in several applications, such as the eplet Registry, EpVix, and commercial software from SAB vendors
Amino Acid Mismatch Score (AAMS)	Online application that compares HLA sequences from the extracellular domain to calculate the number of polymorphic amino acids for a given donor HLA mismatch in the context of recipient HLA^b^
Electrostatic Mismatch Score (EMS3D)	Online application that quantifies differences in tertiary structure and surface electrostatic potential between donor and recipient HLA molecules^c^
HLA-EMMA	Software that calculates the number of solvent-accessible amino acid mismatches between donor and recipients. Solvent-accessible amino acids are defined as residues that are accessible for the B cell receptor and could therefore potentially interact with the B cell receptor, as well as with antibodies^d^
Snowflake	Algorithm that analyses the surface area of mismatched amino acids while taking HLA protein-specific structural disparities into consideration. Recently extended with an algorithm (Snowball) that predicts local ellipsoid protrusion ranking aiming to enhance the accuracy of prediction of solvent accessibility (together “Snow”)^e^
PIRCHE-II (predicted indirectly recognizable HLA epitopes presented by HLA class II)	Online-available *in silico* model that predicts HLA-derived peptides from mismatched HLA that can be presented in self-HLA class II molecules. While HLAMatchmaker, EMS3D, HLA-EMMA and Snow are based on epitopes recognized by B cells, PIRCHE-II quantifies the number of theoretical T cell epitopes presented in HLA class II^f^

^a^ RJ, Duquesnoy. Hum Immunol (2002) 63(5):339–52.

^b^ Kosmoliaptsis et al. Transplantation (2009) 88(6):791–8.

^c^ Mallon et al. J Immunol (2018) 201(12):3780–92.

^d^ Kramer et al. HLA (2020) 96(1):43–51.

^e^ Niemann et al. Front Immunol (2025) 16:1,548,934.

^f^ Geneugelijk et al. Front Immunol (2018) 9:321.

Lastly, the definition of a cutoff value that divides a study population between low risk and high risk, results in the possibility that when this threshold will be applied in a general population, a patient who received a graft bearing a molecular mismatch level below the threshold still received an organ containing a highly immunogenic mismatch that could lead to DSA formation. In fact, even a single amino acid mismatch on foreign HLA is sufficient to induce antibody formation [47, 74, 75]. Additionally, optimal thresholds for risk stratification likely will be population- and transplant center-specific. This is illustrated by a recent study that suggested that previously defined eplet mismatch thresholds need to be adjusted to be applicable to a patient cohort with different immunosuppression (cyclosporin vs. tacrolimus-based) [76]. Therefore, identification of immunogenic - and thus clinically relevant - molecular mismatches in diverse populations remains important for the refinement of post-transplant risk stratification.

In order to take post-transplant risk stratification based on molecular mismatch levels forward, studies are required to investigate whether molecular mismatch load can identify individual patients that can benefit of reduction of immunosuppression. In the retrospective analysis of the CTOT-09 study, HLA-DQ eplet mismatch load >16 predicted dnDSA formation after tacrolimus withdrawal in 5 out of 8 patients. None of the patients with a HLA-DQ eplet mismatch load below the predefined threshold of 16 developed dnDSA [77]. In the CELIMINN trial, HLA class I and HLA-DQ eplet mismatches predicted dnDSA formation in patients that received tacrolimus monotherapy after discontinuation of prednisone and mycophenolate mofetil [78]. Likewise, a recent study demonstrated that in patients treated with mesenchymal stromal cell (MSC) therapy to facilitate tacrolimus withdrawal, HLA-DQ eplet mismatch levels of ≥11 predicted dnDSA formation in 11 out of 21 patients, versus 0 out of 8 patients with an HLA-DQ eplet mismatch load below the threshold [79]. These studies indicate that the negative predictive value of eplet mismatch load for prediction of dnDSA formation after drug weaning is high, but the positive predictive value is low.

Although previous tacrolimus withdrawal trials selected ‘’immune-quiescent’’ or long term stable kidney transplant patients [77, 80], molecular mismatch analysis was not a criterium to select low risk patients. A next step in the investigation of molecular mismatch for risk stratification should be a prospective study that will randomize patients with low immunological risk based on molecular mismatch analysis to a predefined reduction of immunosuppression at a specific timepoint, such as lower tacrolimus trough levels or complete withdrawal of an immunosuppressive drug. As described above, with a high negative predictive value for dnDSA formation, low eplet mismatch load is expected to be a safe biomarker to guide immunosuppressive drug weaning. Primary and secondary outcomes should include dnDSA formation, rejection, fibrosis, graft loss, and adverse effects of immunosuppression such as infections and malignancies, measured during a follow-up period of at least 5 years. Additionally, therapeutic drug monitoring data such as tacrolimus trough levels should be reported to account for potential bias due to differences in immunosuppressive exposure.

Lastly, as opposed to identifying low risk patients for immunosuppression weaning trials, molecular mismatch load could also be used to select high risk patients for studies with rare endpoints, such as antibody-mediated rejection (AMR). Currently, as the incidence for AMR is low, clinical studies investigating this outcome need to include very large numbers of patients to generate enough power for conclusive results. By selecting patients based on high molecular mismatch load, the study population for intervention studies could be enriched for high risk patients, which would facilitate smaller study cohorts [81].

## Conclusion

The clinical application of molecular mismatch analysis in transplantation is promising and has several approaches. However, it is of importance that the fundamentals of HLA-specific antibody biology and epitope-paratope interactions are further elucidated, to unravel the factors that affect the relative immunogenicity of HLA (molecular) mismatches. Excellent quality research in this field requires further development of human HLA-specific monoclonal antibodies, recombinant (and potentially modified) HLA molecules, as well as the curation and analysis of informative sera and cell lines. The collection of large datasets with high-resolution HLA typed transplant recipients and donors, the availability of SAB data for DSA analysis and kidney biopsy data for rejection will aid in further clinical implementation. Furthermore, the time has come for the initiation of prospective studies that investigate the value of HLA molecular mismatch analysis in post-transplant risk stratification for reduction of immunosuppression.
